# Traversing the effects of ploidy changes in different *Eragrostis curvula* genotypes through high‐throughput RNA sequencing

**DOI:** 10.1002/tpg2.70227

**Published:** 2026-03-28

**Authors:** D. F. Santoro, J. Carballo, M. C. Pasten, C. A. Gallo, E. Albertini, V. Echenique

**Affiliations:** ^1^ Dipartimento di Scienze Agrarie, Alimentari e Ambientali Università degli Studi di Perugia Perugia Italy; ^2^ Centro de Recursos Naturales Renovables de la Zona Semiárida (CERZOS–CCT–CONICET Bahía Blanca) Bahía Blanca Argentina; ^3^ Departamento de Agronomía Universidad Nacional del Sur (UNS) Bahía Blanca Argentina; ^4^ Departamento de Ciencias de la Computación Universidad Nacional del Sur (UNS) Bahía Blanca Argentina

## Abstract

Polyploidization has played a key role in plant genome evolution. *Eragrostis curvula* (Schrad.) Ness, a perennial forage grass species of the *Poaceae* family, is an excellent model for investigating genome duplication due to its natural variation in ploidy levels. To explore the transcriptomic consequences of polyploidy, we performed high‐throughput RNA‐Seq on leaf tissue from 10 *E. curvula* genotypes ranging from diploid to octoploid. Differential expression analyses revealed that the number of differentially expressed genes increased with increasing ploidy, suggesting a rewiring of the gene regulatory network. Several putative genes associated with stress tolerance and epigenetic regulation were modulated at higher ploidy levels. Forage digestibility and saccharification efficiency were likely altered at higher ploidy levels, mainly due to the upregulation of putative genes involved in lignin biosynthesis, cell wall remodeling, and polysaccharide metabolism. Our results may reveal a fine‐tuning regulation favoring stress tolerance over forage digestibility. The analysis of the core set of 433 Ploidy_vs_2x DEGs, consistently expressed in a polyploidy‐sensitive manner, revealed upregulation of genes involved in the ubiquitination pathway, stress response, cell wall remodeling, hormonal regulation, and terpenoid biosynthesis. Ploidy‐dependent transcriptional responses were also observed in the patterns of transcription factor families, underlining a different reprogramming of the transcriptional network at each ploidy level. An integrated network‐based approach combining WGCNA and SWIM (SWItchMiner) identified a predicted master regulator target gene putatively associated with increased forage digestibility. Our findings provide valuable insights into the molecular mechanisms underlying polyploidization in *E. curvula*, with implications for breeding strategies to balance stress tolerance and biomass digestibility.

## Core Ideas

- Ploidy‐sensitive genes were uniquely identified at each ploidy level in *Eragrostis curvula* genotypes.
- Novelty at the cellular level might be generated by increasing ploidy levels.
- Ploidy‐dependent variation in the *E. curvula* transcriptomic landscape reflects both effects of genome duplication and the genetic background of each genotype.
- Co‐expression network analysis underlined the involvement of putative genes associated with lignin biosynthesis, cell wall remodeling, epigenetic regulation, and stress responses at higher ploidy levels.
- A trade‐off between increased stress tolerance and reduced forage digestibility likely occurred at higher ploidy levels.
- This study provides genomic resources for future functional genetic studies aimed at balancing stress tolerance and improving biomass digestibility.

## Plain Language Summary

Many crop species carry extra sets of chromosomes (polyploidy), which can generate novelty at the cellular level. Our study investigated the transcriptomic effects of genome doubling in *Eragrostis curvula*, a forage grass that naturally occurs with two to eight sets of chromosomes. Using an RNA sequencing approach, we searched for genes associated with the transition from diploid to polyploid. Many genes related to stress responses, cell wall reprogramming, and epigenetic regulation were modulated upon genome duplication in *E. curvula*. We identified candidate genes that could provide a polyploid advantage under climate change fluctuations while improving forage digestibility. These genes can be used as targets of breeding programs aimed at balancing stress tolerance and biomass digestibility. Overall, our findings show how having extra chromosome sets can generate useful crop diversity, laying the basis for developing more resilient, high‐quality forage grasses.

**Abbreviations**
DEGs: differentially expressed genes
FDR: false discovery rate
GO: gene ontology
KEGG: Kyoto Encyclopedia of Genes and Genomes
Log2FC: log_2_ fold change
PCA: principal component analysis
SWIM: SWItchMiner
TF: transcription factor
WGCNA: weighted gene co‐expression network analysis

## 1 INTRODUCTION

Polyploidy, the condition of harboring more than two sets of homologous chromosomes, is a major driver of plant genome evolution and speciation in angiosperms (Madlung, 2013; Van De Peer et al., 2017; Wood et al., 2009). Polyploidization has played a crucial role in the domestication of crop species by enhancing their genetic diversity and adaptability (Alix et al., 2017; Ha et al., 2009; Heslop‐Harrison et al., 2023; Salman‐Minkov et al., 2016). The main advantages of polyploids are the increased vigor, where the polyploids are superior in one or more characteristics over their progenitors; gene redundancy, which protects polyploids from deleterious mutations, and the capacity to reproduce asexually, among others (Comai, 2005). Even more, increased gene dosage stemming from polyploidization has been shown to provide an evolutionary advantage during shifting environmental conditions through mechanisms such as genome buffering, increased gene expression flexibility, and epigenetic modifications (Tossi et al., 2022; Van de Peer et al., 2021). These structural genome changes give rise to allelic novelty, resulting in unique physiological and morphological traits not found in the diploid progenitors (De Storme & Mason, 2014; Fawcett et al., 2009; Heslop‐Harrison et al., 2023; Vanneste et al., 2014). An increase in cell size associated with genome doubling exerts nucleotypic effects by initiating a complex network of intracellular adjustments, including modifications in chromatin organization, transcriptional regulation, organelle architecture, and intracellular trafficking (Doyle & Coate, 2019; Soltis & Soltis, 2012). This genomic plasticity fosters functional diversification through subfunctionalization and neofunctionalization, enhancing the adaptive potential of polyploid lineages to environmental fluctuations (Morris et al., 2024).

*Eragrostis curvula* (Schrader) Ness, commonly known as weeping lovegrass, is a perennial C4 grass of the *Poaceae* family, subfamily Chloridoideae, native to Southern Africa (Stalker & Wright, 1975). It is mainly used as forage in arid and semi‐arid regions due to its spring‐summer growth, high biomass production, and remarkable adaptability to drought and nutrient‐poor soils. However, its forage quality is limited by several factors, including high fiber content, low crude protein, and the accumulation of silica‐based phytoliths in aerial tissues, significantly reducing digestibility and palatability (Gallardo et al., 2023). Previous studies of in vivo and in vitro digestibility of this grass showed differences in forage quality among tetraploid and between tetraploid and heptaploid cultivars, suggesting that cv. Morpa (4x) has greater digestibility than cv. Tanganyika (4x) and cv. Don Pablo (7x) (Luciani et al., 2012; Voigt et al., 1970). Interestingly, studies carried out on the nutritional quality of different *E. curvula* cultivars for grazing showed that there are not or there are only very few variations in lignin content between the mentioned genotypes (Luciani et al., 2012; Villalba et al., 1991; Voigt et al., 1981), being probably the observed differences in digestibility due to changes in lignin composition. Despite these limitations, *E. curvula* has emerged as an excellent model for investigating genome duplication, since it includes cytotypes with different ploidy levels (2x–8x), displaying different reproductive modes. Diploid genotypes reproduce sexually through cross‐pollination, whereas polyploids exhibit diplosporous apomixis (obligate or facultative) and sexual reproduction (Voigt & Bashaw, 1976; Voigt et al., 2004). Most genotypes evaluated so far across different ploidy levels exhibit bivalent and polyvalent pairing behavior during meiosis (Burson & Voigt, 1996; Poverene, 1988; Vorster, 1977). More recently, using a bioinformatic approach, we have found that in the tetraploid Tanganyika INTA genotype, some chromosomes show mainly autotetraploid behavior, while others show allotetraploid behavior, supporting segmental allotetraploidy.

Recent genomic resources, including a high‐quality reference genome and pangenome assembly, have enabled the identification of genetic loci associated with reproduction, forage quality, and stress resilience, paving the way for molecular breeding and bioengineering strategies aimed at improving both the agronomic performance and nutritive value of this underutilized forage crop (Carballo et al., 2019, 2023). Comparative genomics has also shown a closer relationship between *E. curvula* and other *Poaceae* crops, suggesting shared evolutionary trajectories and potential translational genomics applications (Carballo et al., 2019).

In addition to its agricultural relevance, *E. curvula* is increasingly considered a potential biofuel feedstock. In particular, its high biomass yield, perennial nature, and ability to grow on marginal lands unsuitable for food crops make it an attractive target for bioenergy production, especially in semi‐arid subtropical marginal lands (Carballo et al., 2019; Lauriault et al., 2012). The discovery of genes involved in lignin biosynthesis further supports targeted breeding and genetic engineering strategies to improve both forage digestibility and cell wall degradation efficiency in biofuel conversion processes.

Molecular analyses based on next‐generation sequencing (NGS) have significantly reduced the cost and complexity of developing genomic resources for non‐model species (Wang et al., 2009). This technology enables the comprehensive quantification of gene expression across organs or developmental stages, offering insights into the biological processes and metabolic pathways influenced by specific physiological or genetic contexts. Transcriptomic studies in *E. curvula* have previously been conducted, including gene expression shifts in reproduction, stress response, and epigenetic regulation (Garbus et al., 2019; Pasten et al., 2022; Selva et al., 2020). Integrated transcriptomic and small RNA (sRNA) analyses have unveiled the role of miRNA–mRNA interactions and DNA methylation in regulating gene expression across reproductive transitions, such as the shift from sexuality to apomixis, which involve tightly regulated gene silencing mechanisms modulated by sRNAs and DNA methylation (Garbus et al., 2019). Finally, the genes identified as lost during the diploidization of the genotype Tanganyika INTA in Victoria cv. were explored using a pangenomic approach (Carballo et al., 2023).

This study investigated the transcriptomic landscape of 10 *E. curvula* genotypes with contrasting ploidy levels to identify key genes, enriched functional categories, metabolic pathways, and crucial nodes activated during ploidy transition. An integrated network‐based approach combining weighted gene co‐expression network analysis (WGCNA) and SWItchMiner (SWIM) was used to correlate co‐expression modules and detect crucial nodes activated during ploidy transition. Our findings provide novel insights into the molecular mechanisms shaped by polyploidy and lay the groundwork for future molecular breeding efforts to improve environmental resilience and biomass quality in this underutilized yet promising grass.

**Core Ideas**

- Ploidy‐sensitive genes were uniquely identified at each ploidy level in *Eragrostis curvula* genotypes.
- Novelty at the cellular level might be generated by increasing ploidy levels.
- Ploidy‐dependent variation in the *E. curvula* transcriptomic landscape reflects both effects of genome duplication and the genetic background of each genotype.
- Co‐expression network analysis underlined the involvement of putative genes associated with lignin biosynthesis, cell wall remodeling, epigenetic regulation, and stress responses at higher ploidy levels.
- A trade‐off between increased stress tolerance and reduced forage digestibility likely occurred at higher ploidy levels.
- This study provides genomic resources for future functional genetic studies aimed at balancing stress tolerance and improving biomass digestibility.

## 2 MATERIALS AND METHODS

### 2.1 Plant material and experimental conditions

The plants used in this work were previously described by Carballo, Zappacosta, Selva et al. (2021) and consisted of genotypes with different ploidy levels and reproductive modes. Victoria (2x) and OTA‐S (4x) were used as sexual genotypes, while the apomictic genotypes were grouped by their different ploidy levels: five tetraploids (Bahiense cv., Ermelo cv., Morpa cv., Tanganyika USDA cv., and Tanganyika INTA), the hexaploid Don Eduardo, the heptaploid Don Pablo, and the octaploid Don Juan. Detailed information is provided in Table S1. These genotypes have been maintained at CERZOS‐CONICET, located in Bahía Blanca, Buenos Aires, Argentina (38°43ʹ0ʺ S, 62°16ʹ0ʺ W). The study was conducted in a climate‐controlled greenhouse at 25 ± 4°C under natural light. Three biological replicates for each genotype were produced by tiller propagation. Leaf tissue was collected for all triplicates, immediately frozen in liquid nitrogen, and stored at −80°C until further use. Thirty leaf samples (10 genotypes × 3 biological replicates) were used for RNA sequencing.

### 2.2 RNA extraction

Total RNA was isolated from 30 to 40 mg of ground flash‐frozen leaf tissue using the SV Total RNA Isolation System (Promega), including DNase I treatment according to the manufacturer's instructions. Degradation and contamination were evaluated by 1% agarose gel electrophoresis. Thirty leaf samples (10 genotypes × 3 biological replicates) were used. Purity and concentration were checked using the DeNovix DS‐11 spectrophotometer (DeNovix). Integrity was assessed using the Agilent Bioanalyzer 5400 system (Agilent Technologies). Samples with 260/280 and 260/230 ratios ranging from 1.8 to 2.2 were used for sequencing. RNA integrity number was used to assess quality. Our average of 7.3 indicated that all samples were suitable for sequencing (Table S2).

### 2.3 Library preparation and mapping to the reference genome for RNA‐Seq

The transcriptome sequencing was conducted by Novogene. Libraries were generated on the Illumina NovaSeq X Plus Series to generate PE150 using the NGS RNA Library Prep Set (Novogene Biotech, PT044). The resulting reads were aligned to the reference genome (Carballo et al., 2019) using HISAT2 software (Pertea et al., 2016). Gene expression levels were estimated using the GenomicAlignments R library (Lawrence et al., 2013), based on read alignments stored in the resulting BAM files and the reference genome annotation (Carballo et al., 2019).

### 2.4 Quantification of gene expression and differential expression analysis

**FIGURE 1:**
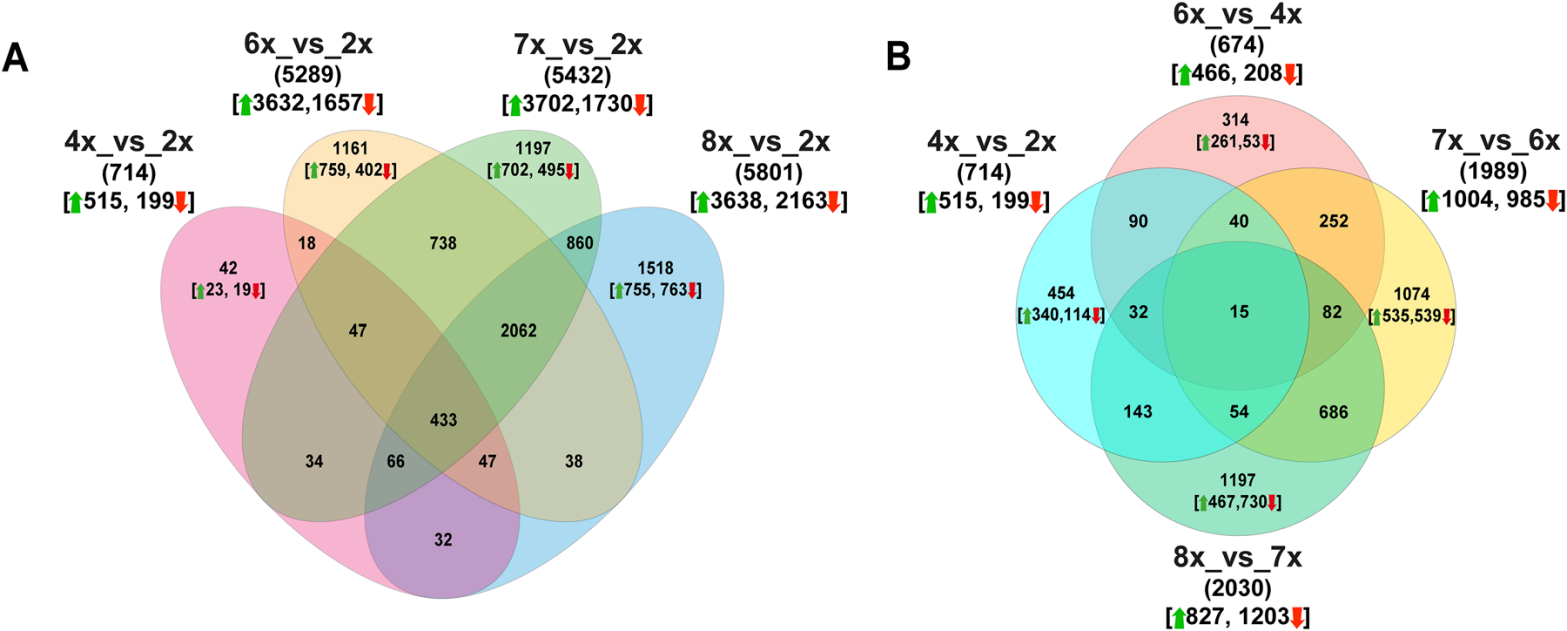
Differentially expressed gene (DEG) analysis in *E. curvula*. (A) Venn diagram of DEGs at different ploidy levels. The upward green arrow represents the upregulated genes, while the downward red arrow represents the downregulated genes. (B) Number of DEGs between different ploidy levels. An lfcThreshold of 1 with a *p*_adj_ ≤ 0.05 was adopted.

Read counts were used for DESeq2 (1.26.0 version) to identify differentially expressed genes (DEGs) (Love et al., 2014). The resulting *p*‐values were adjusted using Benjamini and Hochberg's approach to control the false discovery rate (FDR). Hierarchical clustering and principal component analysis (PCA) were performed based on the normalized counts. The phylogenetic relationship among *E. curvula* genotypes was reconstructed. An adjusted *p*‐value cutoff of 0.05 and an absolute log_2_ fold change (Log_2_FC) threshold of 1 were used to identify significantly DEGs. To identify ploidy‐sensitive DEGs, the genotypes were compared pairwise to avoid unbalanced comparisons, and then a Venn diagram was produced to identify DEGs in common between the comparisons. We investigated four comparisons across different ploidies: tetraploids versus diploids (4x_vs_2x), hexaploids versus diploids (6x_vs_2x), heptaploids versus diploids (7x_vs_2x), and octaploids versus diploids (8x_vs_2x) (Figure 1A). Effects of genome doubling on gene expression levels were also evaluated at increasing ploidy levels (4x_vs_2x, 6x_vs_4x, 7x_vs_6x, and 8x_vs_7x) (Figure 1B). A gene‐wise linear mixed‐effects model explicitly partitioning variance attributable to ploidy versus genotype was constructed using the variancePartition package (Hoffman & Schadt, 2016) (https://bioconductor.org/packages/release/bioc/html/variancePartition.html). Ploidy, genotype, and the interaction of ploidy × genotype terms were treated as random effects in a linear mixed model. Variance fractions were extracted on a per‐gene basis to estimate genome‐wide effects.

### 2.5 Gene ontology, Kyoto Encyclopedia of Genes and Genomes enrichment, and iTAK analysis

We conducted gene ontology (GO) functional enrichment analysis on all DEGs (up‐ and down‐regulated) that were uniquely found in each group with AmiGO 2 (version 2.5.17) (Carbon et al., 2009). The full set of DEGs identified in each comparison was separately submitted to KOBAS (version 3.0, corrected *p*‐value ≤ 0.05) to identify significantly enriched pathways in the Kyoto Encyclopedia of Genes and Genomes (KEGG) database (Bu et al., 2021). Orthology‐based annotation was performed using *Arabidopsis thaliana* as the reference species, as it provides the most comprehensive and well‐curated functional and pathway annotations for non‐model species. Enrichment *p*‐values were first calculated using Fisher's exact test. The resulting *p*‐values were adjusted using Benjamini and Hochberg's approach to control the FDR. An adjusted *p*‐value of 0.05 and an absolute Log_2_FC of 1 were implemented to filter the over‐ and under‐represented categories. Gene functional enrichment was conducted using MapMan 3.6.0RC1 (https://mapman.gabipd.org/) (Thimm et al., 2004). DEGs were mapped using Mercator4 (7.0 version) (Lohse et al., 2014) to classify and predict functions, and the resulting maps were analyzed with MapMan. Significant DEGs (*p*_adj_ ≤ 0.05) and their respective Log_2_FC values were used for alignment with the Mercator map. iTAK (Plant Transcription Factor & Protein Kinase Identifier and Classifier) was used to identify DEGs' transcription factor (TF) families (Zheng et al., 2016). An adjusted *p*‐value cutoff of 0.05 and an absolute Log_2_FC threshold of ±1 were used to filter the significantly up‐ and down‐regulated genes. To identify TF families significantly enriched at each ploidy level relative to their genome‐wide abundance, enrichment analyses were performed using one‐sided Fisher's exact tests with a *p*‐value ≤ 0.05.

### 2.6 Building gene co‐expression networks

Co‐expression network analysis identified gene clusters (modules) with highly correlated expression profiles (hub genes) using the weighted gene co‐expression network analysis (WGCNA) package in R (Langfelder & Horvath, 2008) (https://github.com/Danilofabrizio92/WGCNA.git). The read counts matrix was filtered by retaining genes with more than 10 alignments in at least three samples. Data were normalized using the DESeq2 median of ratios method (Love et al., 2014), which set ploidy as a linear model variable. Scale‐free topological analysis using the *pickSoftThreshold* function of WGCNA chose the proper soft‐thresholding power (Langfelder & Horvath, 2008). We selected the value closest to 0.9 to ensure a scale‐free co‐expression network when the soft threshold ranged from 1 to 20. A weighted adjacency matrix was constructed on the normalized data using the automatic module detection function *blockwiseModules* of WGCNA (Langfelder & Horvath, 2008) with the following parameters: net_type = signed, minModuleSize = 30, mergeCutHeight = 0.25, deepSplit = 2, corType = Pearson, randomSeed = 42, and power = 18. Identified modules were corrected using a *k*‐means clustering analysis with the *applyKMeans* function of *CoExpNets* (Botía et al., 2017). We used module eigengenes and gene significance to retrieve modules associated with specific ploidy‐dependent experimental conditions.

SWIM integrated network analysis was used to further screen significant hub genes (https://github.com/sportingCode/SWIMmeR.git) (Paci et al., 2017) with an unweighted correlation network to identify predicted master regulators associated with changes in the transcriptome. SWIM‐based correlation network analysis was applied to predict genes affected by ploidy levels. An adjusted *p*‐value cutoff of 0.05 and a Log_2_FC threshold of ±1 were used to search for significant nodes based on Pearson correlation.

## 3 RESULTS

### 3.1 Sequencing and quantification

A total of 2155 million paired‐end reads with an average of 71.83 million paired‐end reads (approximately 10.77 Gb) per sample were retained (Table S2). The average percentages of Q30 and guanine/citosine were 96.14% and 53.33%, respectively. The filtered reads were aligned to the reference genome, and transcript abundance was quantified using the reference genome annotation, which comprises 54,589 transcripts. Hierarchical clustering showed high similarity among the three biological replicates within each group, indicating good sample reproducibility (Figure S1A). PCA analysis was used to assess transcriptome differences among groups. The results showed distinct clustering across different ploidy levels (Figure S1B). Interestingly, the PCA clustering was ploidy‐aware. Samples belonging to 4x effectively clustered together (Figure S1B). A clear separation at higher ploidy levels was observed (Figure S1B). Within this region, 6x and 7x genotypes formed an adjacent cluster, while 8x remained relatively close to the 6x and 7x groups, suggesting some degree of transcriptomic divergence (Almeida‐Silva & Van De Peer, 2025). Expression‐based hierarchical clustering further clarified these relationships, indicating that 7x and 8x genotypes are more closely related to each other (Figure S1C).

### 3.2 Identification of DEGs

The *E. curvula* transcriptional response was characterized by identifying the genes whose expression levels changed at different ploidy levels (Figure 1). We filtered out genes with low expression levels by setting the threshold for the minimum number of counts to 10 in at least three samples, resulting in the retention of 35,440 transcripts of the initial 54,589 across the entire dataset. Differential gene expression analysis was conducted by comparing individual polyploid genotypes to the diploid Victoria (2x) counterpart to identify unique and shared genes associated with increased ploidy relative to the 2x reference. An increased number of DEGs was observed at increasing ploidy levels, suggesting a rewiring of the transcriptional network upon polyploidization (Figure 1A; Table S3A). A total of 42 genes (23 up‐ and 19 down‐regulated) were unique to the 4x_vs_2x group (pink circle in Figure 1A), while 1161 genes (759 up‐ and 402 down‐regulated) were unique to the 6x_vs_2x group (yellow circle in Figure 1A). Individual genotypes at the 4x level exhibited stochastic differences, with a higher prevalence of DEGS in Tanganyika USDA (Table S3A). A majority of DEGs were found at higher ploidy levels, with 1197 unique genes in the 7x_vs_2x group (702 up‐ and 495 down‐regulated, green circle) and 1518 in the 8x_vs_2x (755 up‐ and 763 down‐regulated, blue circle) (Figure 1A). Interestingly, an increased number of uniquely expressed genes in the 8x_vs_2x group were downregulated (763) compared to the other groups. These findings suggest that transcriptome reprogramming occurs with increasing ploidy, thereby modifying gene expression levels in response to genome duplication. We also identified a core set of 433 Ploidy_vs_2x DEGs consistently expressed in a polyploidy‐sensitive manner (Figure 1A; Table S3B). Among these genes, 430 (334 up‐ and 96 down‐regulated) exhibited the same pattern of expression levels in all the polyploid genotypes compared to the 2x counterpart, while three genes were upregulated in 4x_vs_2x, 6x_vs_2x, and 7x_vs_2x and downregulated in 8x_vs_2x. Within this set of genes, we observed the upregulation of many *nucleotide binding site leucine‐rich repeat* (*NBS‐LRR*) genes associated with biotic stress tolerance in polyploid genotypes compared to the 2x_counterpart (Table S3B). Homologs of ethylene‐responsive genes were also found to be upregulated. During epigenetic‐responsive gene searches, we observed upregulation of genes associated with plant growth and development, including zinc finger BED domain‐containing protein *DAYSLEEPER* and *MAIN‐LIKE 1* (Bundock & Hooykaas, 2005; Ühlken et al., 2014). It is worth mentioning that a homolog gene encoding for *DYAD*, which is required for meiotic chromosome organization and female meiotic progression (Agashe et al., 2002), was found to be upregulated. Homologs of *beta‐1,6‐galactosyltransferase* *GALT29A*, *omega‐hydroxypalmitate O‐feruloyl transferase*, and probable *glucomannan 4‐beta‐mannosyltransferase 1* were also found to be upregulated, which in turn may influence forage quality and digestibility in polyploid genotypes (Table S3B). In addition, three homologs of the senescence‐specific cysteine protease *SAG12* were also upregulated. The dissection of secondary metabolism genes revealed upregulation of a homolog of anthocyanidin reductase, a key enzyme in proanthocyanidin biosynthesis, compounds considered an important trait influencing forage quality (Xie et al., 2006). Interestingly, using 2x as a reference, we observed that DEGs uniquely present in 4x do not significantly increase, whereas in 6x, 7x, and 8x, the number of genes increases by an average of 1292 genes with increasing ploidy.

The effect of genome doubling on gene expression was also assessed by comparing transcriptomic profiles across increasing ploidy levels: 4x_vs_2x, 6x_vs_4x, 7x_vs_6x, and 8x_vs_7x. The percentage of DEGs in the 4x_vs_2x and 6x_vs_4x groups was considerably low, ranging from 1.90% (674 DEGs) to 2.01% (714 DEGs) of all 35,440 genes (Figure 1B; Table S3). On the contrary, around 5.67% of DEGs were detected at increased ploidy levels, with 1989 genes in 7x_vs_6x and 2030 genes in 8x_vs_7×1989, respectively (Figure 1B; Table S3). However, a consistent trend was observed in the ratio of upregulated to downregulated genes: most DEGs at the highest ploidy level were downregulated rather than upregulated, reflecting partial dosage compensation of gene expression at the 8x level (Figure 1B; Table S3). This finding is consistent with the ratio of up‐ to down‐regulated genes observed across different ploidies (Figure 1A; Table S3).

To assess the fraction of total gene expression variance explained by ploidy and genotype, we fitted a gene‐wise linear mixed model using the variancePartition (Hoffman & Schadt, 2016). Variance partitioning was assessed over the ploidy, genotype, and the interaction between ploidy and genotype. Even though the change of expression in many genes was not explained by the factors assessed here, the interaction of ploidy:genotype showed an increased number of genes with a high percentage of the variance explained by the gene expression compared with ploidy and genotype alone (Figure S2).

### 3.3 Functional classification of GO terms affected by polyploidization

**FIGURE 2:**
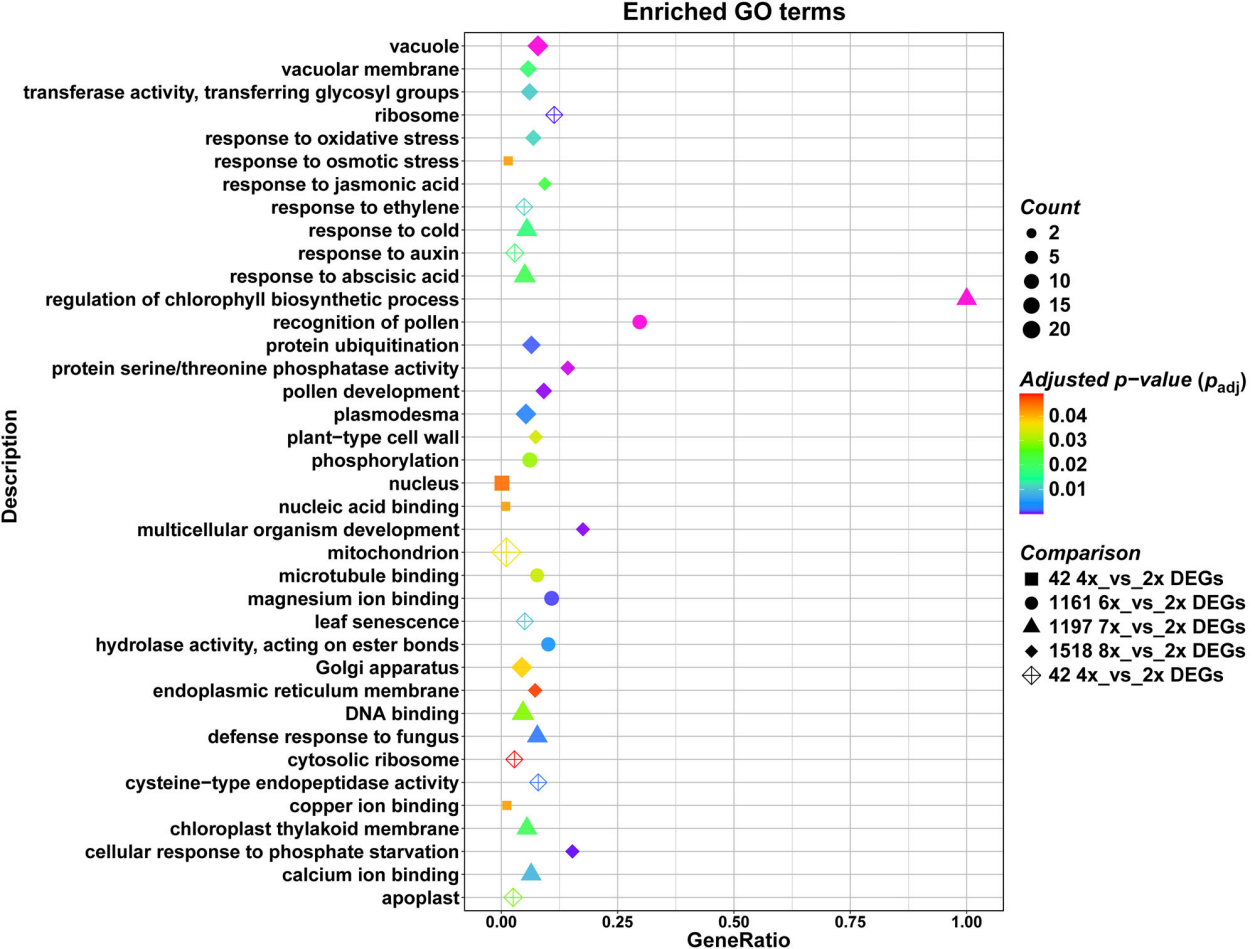
Distribution of the most enriched gene ontology (GO) terms across ploidy levels. The enriched GO categories are displayed on the *Y*‐axis, while the *X*‐axis shows the gene ratio, defined as the proportion of genes from the input dataset associated with each GO term relative to the total number of input genes. The color of the GO terms is represented by a gradient from blue to red according to the adjusted *p*‐value, with red indicating higher statistical significance. The size of the symbols corresponds to the number of genes associated with each GO category.

Ploidy‐specific transcriptional differences were further investigated through an enrichment analysis of GO terms. Polyploidy exhibited different transcriptional consequences at each ploidy level (Table S4). In the 42 4x_vs_2x DEGs, the most unique enriched GO terms were nucleus, response to osmotic stress, copper ion binding, and nucleic acid binding (Table S4, filled squares in Figure 2). Phosphorylation, magnesium ion binding, recognition of pollen, calmodulin binding, hydrolase activity, acting on ester bonds, and microtubule binding were the most unique GO terms found in the 1161 6x_vs_2x DEGs (Table S4, filled circles in Figure 2). Among the most enriched GO terms uniquely identified in the 1197 7x_vs_2x DEGs, DNA binding, response to abscisic acid, response to cold, chloroplast thylakoid membrane, defense response to fungus, calcium ion binding, and regulation of chlorophyll biosynthetic process were observed (Table S4, filled triangles in Figure 2C). In the 1518 8x_vs_2x DEGs, Golgi apparatus, vacuole, plasmodesma, protein ubiquitination, transferase activity, transferring glycosyl groups, vacuolar membrane, pollen development, and response to oxidative stress were identified among the most unique GO terms (Table S4, filled diamonds in Figure 2). Moreover, protein serine/threonine phosphatase activity, plant‐type cell wall, endoplasmic reticulum membrane, cellular response to phosphate starvation, multicellular organism development, and response to jasmonic acid were also detected at the 8x level (filled diamonds in Figure 2). The functional annotation of the group of all genes differentially expressed between polyploid and diploid genotypes (433 Ploidy_vs_2x DEGs) revealed a core gene set of unique GO terms deregulated in a ploidy‐independent manner (GO terms shared among all polyploid genotypes) associated with mitochondrion, response to auxin and apoplast, followed by ribosome, cysteine‐type endopeptidase activity, leaf senescence, response to ethylene, and cytosolic ribosome (Table S4, empty diamonds in Figure 2).

### 3.4 Metabolic pathways related to ploidy levels in *E. curvula*

**FIGURE 3:**
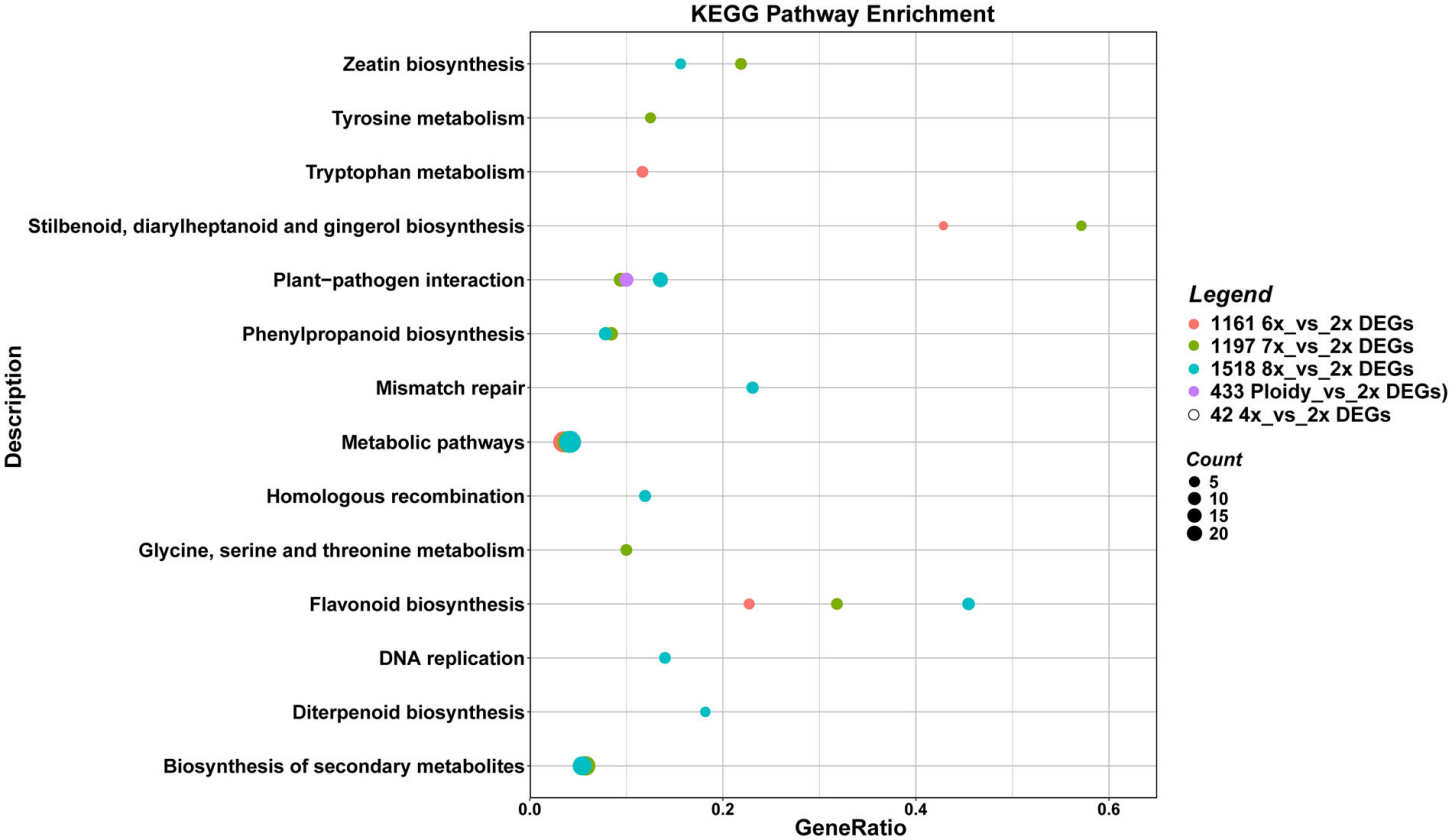
Kyoto Encyclopedia of Genes and Genomes (KEGG) enrichment analysis for the unique differentially expressed genes (DEGs) found in the five groups (42 4x_vs_2x DEGs, 1161 6x_vs_2x DEGs, 1197 7x_vs_2x DEGs, 1518 8x_vs_2x DEGs, and 443 Ploidy_vs_2x DEGs). The enriched KEGG categories are listed, and the proportion of genes from the input dataset associated with each pathway relative to the total number of input genes is reported on the *X*‐axis (gene ratio).

The set of unique DEGs retrieved from the four groups depicted in Figure 1A was mapped onto the KEGG database to detect ploidy‐responsive metabolic pathways. The distribution of KEGG pathways is shown in Figure 3. Focusing on differences between different ploidy levels versus 2x, no significant pathways were found in the 42 4x_vs_2x DEGs. “Stilbenoid, diarylheptanoid, and gingerol biosynthesis” pathway overlapped with 1161 6x_vs_2x DEGs and 1197 7x_vs_2x DEGs (paired red and green dots in Figure 3), whereas the “tryptophan metabolism” pathway was uniquely enriched in the 1161 6x_vs_2x DEGs (isolated red dot in Figure 3). Considering the 1197 7x_vs_2x DEGs, “tyrosine metabolism” and “glycine, serine, and threonine metabolism” pathways were observed (isolated green dots in Figure 3). “Metabolic pathways” and “flavonoid biosynthesis” pathways were found in common among 1161 6x_vs_2x DEGs, 1197 7x_vs_2x DEGs, and 1518 8x_vs_2x DEGs (paired red, green, and cyan dots in Figure 3). Among the enriched KEGG terms found in common between 1197 7x_vs_2x DEGs and 1518 8x_vs_2x DEGs, “zeatin biosynthesis,” “phenylpropanoid biosynthesis,” and “biosynthesis of secondary metabolites” were identified (paired green and cyan dots in Figure 3). “Mismatch repair,” “homologous recombination,” “DNA replication,” and “diterpenoid biosynthesis” were exclusively identified in 1518 8x_vs_2x DEGs (isolated cyan dots in Figure 3). Considering the 433 Ploidy_vs_2x DEGs shared among all groups, only the “plant–pathogen interaction” pathways were identified (isolated violet dot in Figure 3).

To gain further insight into the pathways influenced by polyploidization, the MapMan approach (version 3.6.0RC1) was applied (Table S5). MapMan analyses revealed the involvement of flavonoid biosynthesis alongside a notable downregulation of genes associated with RNA biosynthesis and protein homeostasis within the 42 4x_vs_2x DEGs (Table S5A). The 1161 6x_vs_2x DEGs exhibited a significant activation of core photosynthetic processes, including carbon fixation and repair mechanisms, coupled with a strong upregulation of genes associated with hormone biosynthesis and chromatin remodeling (Table S5B). We also observed the upregulation of many genes related to carbohydrate and lipid metabolism. Additionally, increased biosynthesis of secondary metabolites and a robust response to oxidative stress were also identified in this group.

In contrast, the 1096 7x_vs_2x DEGs revealed a significant metabolic reprogramming, marked by a downregulation of core genes associated with both photosynthesis electron transport, Calvin cycle enzymes, and photorespiration, as well as components of glycolysis. Notably, genes involved with auxin transport and gibberellin biosynthesis were downregulated, indicating a hormonal imbalance at the 7x ploidy level (Table S5C). The ubiquitin‐proteasome system was found to be activated due to the upregulation of *E3 ubiquitin ligase*‐related genes. Interestingly, a remarkable increase in the *glutathione S‐transferase* (*GST*) gene was observed (Table S5C). Notably, a profound re‐adjustment of the transcriptional machinery occurred at the highest ploidy level (1518 8x_vs_2x DEGs). We observed the upregulation of genes involved in photosynthetic assembly machinery, including *chaperonin‐like* *RBCX protein* 1 (*RBCX1*) and *chlororespiratory reduction 3* (*CRR3*) (Table S5D). A near‐complete suppression of growth‐promoting hormones was observed, alongside the upregulation of abscisic acid‐ and ethylene‐related genes (Table S5D). This metabolic shift was further corroborated by extensive chromatin remodeling, RNA biosynthesis and protein homeostasis changes, and a significant upregulation of terpene and flavonoid synthesis enzymes (Table S5D). Focusing on the 443 Ploidy_vs_2x DEGs shared by all the comparisons (set of genes consistently sensitive to ploidy level, Figure 1A), we found the upregulation of the *13‐lipoxygenase* (*13‐LOX*) gene, known to be involved in wound response (Glauser et al., 2009) (Table S5E). Many genes associated with RNA biosynthesis and homeostasis were strongly upregulated (Table S5E). We also observed the upregulation of genes involved in the ubiquitination pathway, such as *SUMO*, *FBL*, *DSK2*, and *DAR*, suggesting an ongoing protein turnover and post‐translational regulation in polyploid genotypes. An upregulation of *beta‐1,6‐galactosyltransferase* (*GALT29A*) was observed, suggesting enhanced cell wall remodeling leading to more recalcitrant biomass (Dilokpimol et al., 2014). Additionally, genes encoding for enzymes involved in terpenoid biosynthesis were found to be either up‐ or down‐regulated at the highest 8x ploidy level.

### 3.5 Identification of TF families

**FIGURE 4:**
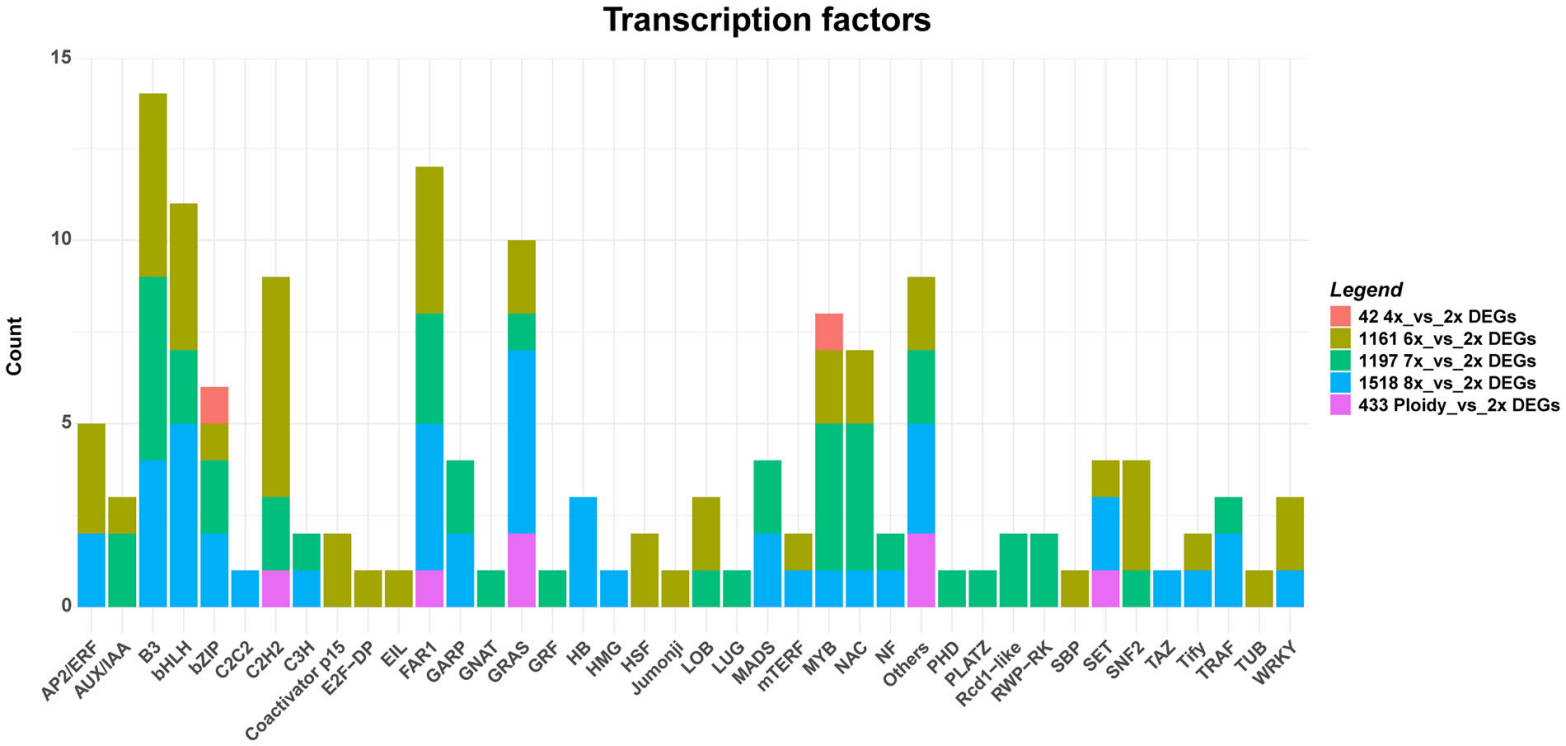
Distribution of transcription factor (TF) families among genes responsive to polyploidization. Each bar represents the number of genes belonging to each transcription factor family.

We assessed the effect of the ploidy level on TF expression by examining the number of TFs present within the four groups of genes defined above (Figure 1). Differential expression analysis revealed specific TF families modulated across ploidy levels (Figure 4). In the 42 4x_vs_2x DEGs, homologs of *VIP1‐2* and *RL1* were downregulated (Table S6A). Fifty‐one (38 up‐ and 13 down‐regulated) TF genes were observed in the 1161 6x_vs_2x DEGs. Looking at the most upregulated TF genes, four homologs of *FAR1‐RELATED SEQUENCE 5* (*FRS5*), a key regulator of plant growth and development (Ma & Li, 2018), were identified (Table S6B). In addition, a homolog of *E2F TF‐like E2FE* (*E2FE*), known to act as a transcriptional repressor of endoreduplication (Radziejwoski et al., 2011), was also detected among the upregulated TF genes. Upregulation was also observed for genes involved in chromatin remodeling, including *ISWI chromatin‐remodeling complex ATPase CHR17* (*CHR17*), *histone‐lysine N‐methyltransferase ASHH3* (*ASHH3*), and *paired amphipathic helix protein Sin3‐like 2* (*SNL2*) (Table S6B). Additionally, two homologs of ethylene‐responsive gene (*ERF073*, *EIL3*), alongside two homologs of *NAC domain*‐*containing protein 74* (*NAC074*), were upregulated. In the 1197 7x_vs_2x DEGs, 45 (26 up‐ and 19 downregulated) TF genes were identified. Among them, we found the upregulation of *two‐component response regulator* *ARR3* (*ARR3*), *B3 domain‐containing protein REM8* (*REM8*), and *histone deacetylase HDT3* (*HDT3*) (Table S6C). Consistent with the 1,161 6x_vs_2x DEGs, a homolog of *FRS5* was also detected among the upregulated TF genes. Auxin‐responsive proteins *IAA26* and *IAA31* were also found to be upregulated. Hormonal regulation was further supported by the upregulation of homologs of *two‐component response regulator* *ARR12* and *growth‐regulating factor 5* (*GRF5*), both involved in cytokinin signaling (Ishida et al., 2008; Vercruyssen et al., 2015). A member of the lateral organ boundaries domain (*LBD36*) involved in developmental boundary formation and organogenesis (Chalfun‐Junior et al., 2005) was upregulated. Moreover, a homolog of *BPM3*, which mediates the proteasomal degradation of target proteins, was also upregulated. The phytochrome‐interacting TF *PIF3* was upregulated, suggesting a positive role in light‐mediated chloroplast development (Monte et al., 2004). Considering the 1518 8x_vs_2x DEGs, 46 (25 up‐ and 21 down‐regulated) TF genes were retrieved (Table S6D). Consistent with the previous results, a homolog of *FRS5* was upregulated along with homologs of protein *FAR1‐RELATED SEQUENCE 6* (*FRS6*) and protein *FAR1‐RELATED SEQUENCE 10* (*FRS10*) (Table S6D). Two homologs of *BTB/POZ* and *MATH domain‐containing protein 1* (*BPM1*) were upregulated, suggesting the pivotal role of ubiquitination‐dependent proteasomal degradation in polyploid plants (Martelotto et al., 2005). A homolog of *homeobox‐leucine zipper protein* *PROTODERMAL FACTOR 2* (*PDF2*) and two homologs of *homeobox protein knotted‐1* (*KNAT6* and *KNAT1*), functioning as positive regulators of embryo development (Belles‐Boix et al., 2006; Ogawa et al., 2015; Truernit & Haseloff, 2008), were also found to be upregulated. These findings are supported by the upregulation of *axial regulator YABBY 1* (*YAB1*), essential for meristem maintenance and other biological processes (Zhang et al., 2020). Furthermore, we observed the upregulation of *histone acetyltransferase HAC1* (*HAC1*) and *histone‐lysine N‐methyltransferase SUVH1* (*SUVH1*), consistent with the role of polyploidization in chromatin architecture modification (Tossi et al., 2022; Van de Peer et al., 2021). A homolog of *ARR2* was also observed to be upregulated, supporting its role as an enhancer to activate target genes (Sakai et al., 2000). Focusing on the 443 Ploidy_vs_2x DEGs in common among different ploidies, we identified seven (four up‐ and three downregulated) TF genes (Table S6E). Among these TF genes, a homolog of *zinc finger protein* *GAI‐ASSOCIATED FACTOR 1* (*GAF1*), a positive regulator of gibberellin, was upregulated. A putative *ubiquitin‐like‐specific protease 1B* (*ULP1B*) was also upregulated, underlining the role of protein turnover in polyploidy. Notably, a homolog of *protein argonaute 9* (*AGO9*), which is involved in post‐transcriptional regulation, was also observed (Table S6E). Significantly enriched TF families at each ploidy level were further investigated through one‐sided Fisher's exact tests (*p* ≤ 0.05). The results showed that *SNF2*, *B3*, *C2H2*, *coactivator p15*, and *HSF* were significantly associated with the 1161 6x_vs_2x DEGs (Table S6F). In contrast, *B3*, *RWP‐RK*, and *Rcd1‐like* were significantly enriched in the 1197 7x_vs_2x DEGs (Table S6F). At the highest ploidy level (1518 8x_vs_2x DEGs), *GRAS* and *B3* were the only TF families significantly enriched (Table S6F). Looking at the core set of genes significantly associated with polyploidization (433 Ploidy_vs_2x DEGs), the *GRAS* family was exclusively found, underscoring its potential role in polyploid genome evolution and adaptation (Liu et al., 2021). The *GRAS* gene family of TFs plays important roles in many biological processes, such as signal transduction, disease resistance and stress tolerance, and plant growth and development (Lu et al., 2024).

### 3.6 Co‐expression network analysis

**FIGURE 5:**
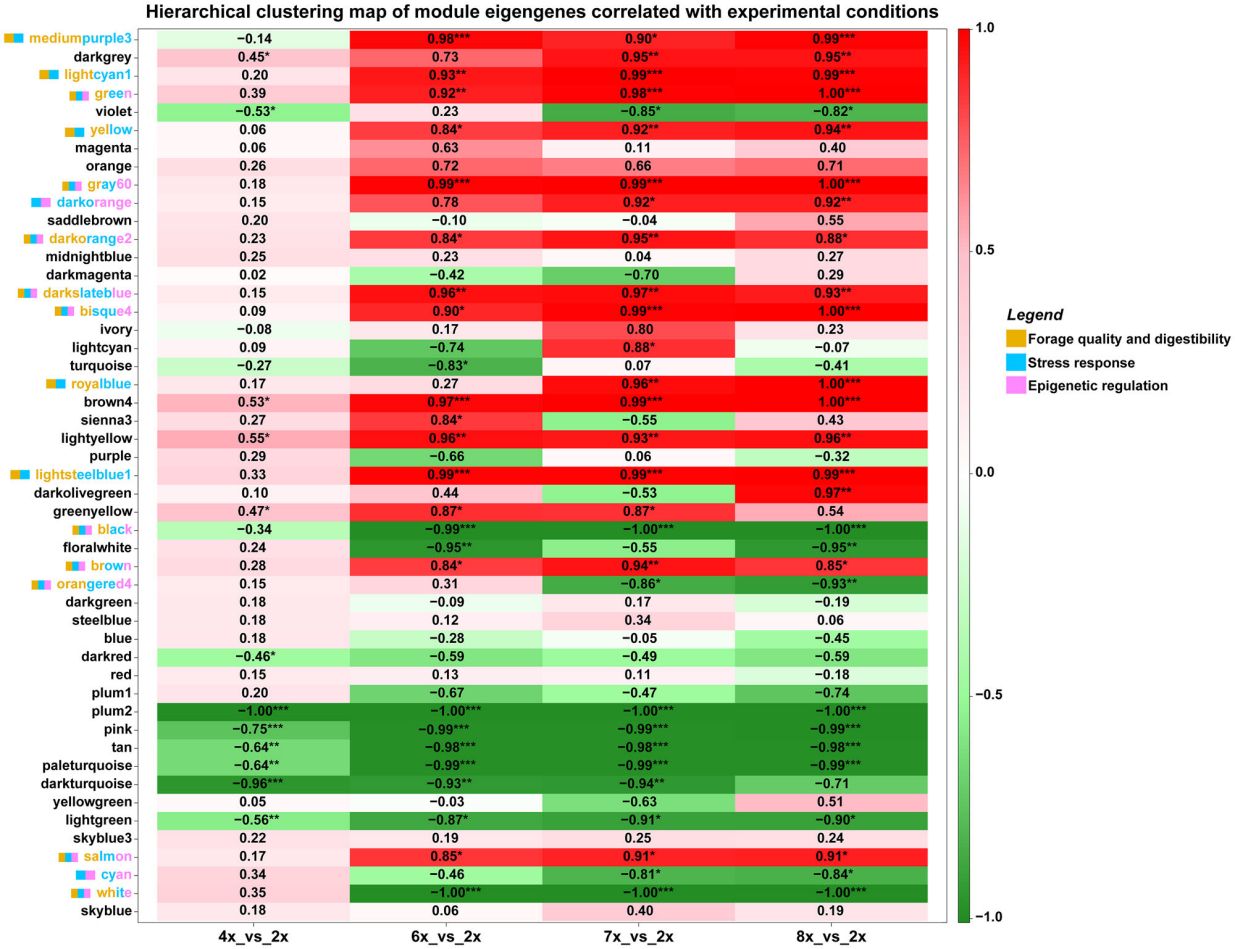
Correlation‐based heatmap of module eigengenes (MEs) obtained after the *k*‐means clustering analysis in the four groups. MEs represent the first principal component of the expression profiles of all genes within a module. Correlations between MEs and groups are calculated using a student's *t*‐distribution approach. Modules with a correlation *p*‐value ≤ 0.05 are marked with asterisks. The color gradient indicates the strength and direction of the correlation: green shades indicate negative correlations, while red or pink shades indicate positive correlations.

A weighted gene co‐expression network analysis (WGCNA R package) was conducted to identify modules that respond to genome duplication in a ploidy‐sensitive manner (Figure 5). We filtered genes with low expression levels (see above), resulting in retention of 35,440 genes across the entire dataset. These were grouped into 49 co‐expression modules through *k*‐means clustering analysis (Figure S3). In WGCNA, two key metrics were evaluated within each module: module membership, which quantifies the extent to which a gene's expression pattern aligns with the module eigengene, and gene significance, which reflects the degree of association between a gene and a specific condition (Langfelder & Horvath, 2008).

Correlation analysis across the 49 modules and the four comparisons (Figure 5) revealed several modules showing consistent correlations with ploidy‐dependent response (4x_vs_2x, 6x_vs_2x, 7x_vs_2x, and 8x_vs_2x groups), either positive (brown4 and lightyellow) or negative (plum2, pink, tan, pale turquoise, dark turquoise, and lightgreen) (Table S7A). WGCNA also showed modules significantly correlated with each group (Figure 5). Within the ploidy‐associated modules that were significantly modulated at higher ploidy levels (mediumpurplue3, lightcyan1, green, yellow, gray60, darkorange, darkorange2, darkslateblue, bisque4, royalblue, lightstellblue1, black, brown, orangered4, salmon, cyan, and white, Figure 5), we observed the involvement of genes implicated in lignin biosynthesis and cell wall remodeling (orange bar, Figure 5), defense response (light blue bar, Figure 5), and epigenetic regulation (pink bar, Figure 5) (Table S7A). Focusing on putative genes associated with forage quality and digestibility, homologs of *shikimate O‐hydroxycinnamoyltransferase* (*HST*), *cinnamyl alcohol dehydrogenase 7* (*CAD7*), and *beta‐glucosidase 46* (*BGLU46*) were upregulated. We also detected the upregulation of *xyloglucan endotransglucosylase/hydrolase protein 32* (*XTH32*), *beta‐galactosidase 14* (*BGAL14*), and *beta‐glucosidase 10* (*BGLU10*), alongside downregulation of *rhamnogalacturonate lyase family protein* (*F21M12.28*) and *alpha‐L‐arabinofuranosidase 2* (*ASD2*). Consistent with the upregulation of WAT1‐related protein (*WAT1*), which is essential for secondary wall formation in fibers (Ranocha et al., 2010), these results may indicate that the forage digestibility and saccharification efficiency are compromised at higher ploidy levels in *E. curvula*.

Several genes associated with biotic and abiotic stress response were also significantly correlated with increased ploidy levels. Disease resistance genes and pattern‐recognition receptors were found to be upregulated (Table S7A). *bZIP*‐related genes associated with pathogen‐associated molecular patterns (*TGA9*) and systemic acquired resistance (*TGA7*) were also upregulated. These results are reinforced by the upregulation of *phospholipase D alpha* (*PLDALPHA1* and *PLDALPHA2*), along with two homologs of *12‐oxophytodienoate reductase 2* (*OPR2*). Reactive oxygen species scavenging machinery was modulated at higher ploidy levels because of the upregulation of *respiratory burst oxidase homolog protein C* (*RBOHC*), *superoxide dismutase [Cu‐Zn] 2* (*CSD2*), peroxidases (*PER34* and *PER35*), and *glutathione S‐transferase U19* (*GSTU19*). ABA‐inducible expression of *dehydrin Rab18* (*RAB18*) was also detected among the upregulated genes. Moreover, a homolog of aquaporin *PIP2‐2* was upregulated. Remarkably, *E3 ubiquitin‐protein ligase* genes, including *NPR5*, *NPR3*, *CHIP*, *SKP1‐like protein 20*, and *SINA‐like 10*, were upregulated (Table S7A).

Epigenetic‐responsive genes associated with DNA methylation and epigenomic changes were also specifically upregulated at higher ploidy levels. Homologs of *DNA‐directed RNA polymerase IV subunit 1* (*NRPD1*), factor of *DNA methylation 1* (*FDM1*), *histone‐lysine N‐methyltransferase EZA1* (*EZA1*), *methyl‐CpG‐binding domain‐containing protein 13* (*MBD13*), and YTH domain‐containing protein ECT3 (*ECT3*) were detected (Table S7A). Interestingly, a homolog of *PHD finger protein ALFIN‐LIKE 2* (*AL2*), which binds H3K4me3 and promotes H2A.Z deposition through the SWR1 complex (Xu et al., 2025), was upregulated.

SWIM analysis was further conducted to identify candidate master regulators within those modules consistently correlated with ploidy‐sensitive expression patterns. Switch genes have been defined as a new class of hub genes characterized by a pronounced negative correlation with the expression profiles of their nearest neighbors, and encode key regulators potentially involved in transcriptional state transitions (Palumbo et al., 2014). A total of 8476 nodes were identified, of which 388 were classified as switch genes (Table S7B). Searching for predicted master regulators associated with cell wall modifications, we identified downregulation of the endoglucanase *GH9B16*, which emerged as a potential switch gene given the established role of *GH9B* endoglucanases in enhancing saccharification efficiency by decreasing cellulose microfibrils (Buchanan et al., 2012; Huang et al., 2019). In addition, a homolog of *laccase‐7* (*LAC7*), previously suggested to play a key role in regulating lignin deposition and secondary xylem development in *Arabidopsis* (Zhao et al., 2015), was identified among the downregulated predicted switch genes (Table S7B).

## 4 DISCUSSION

Polyploidy, the condition of harboring more than two sets of homologous chromosomes, is a major driver of plant genome evolution, influencing key traits such as stress tolerance, biomass accumulation, and reproductive strategies. *Eragrostis curvula* (Schrad.) Ness, a perennial grass of the *Poaceae* family, serves as an excellent model for investigating genome duplication due to its natural variation in ploidy levels. To explore the transcriptional consequences of polyploidization at different ploidy levels, we performed high‐throughput RNA‐Seq on leaf tissue from 10 *E. curvula* genotypes ranging from diploid to octaploid. This approach allows the evaluation of gene expression avoiding the typical mistakes and problems related to primer design and housekeeping genes required to have high‐confidence qPCRs, mainly when very heterozygous genotypes are involved. The genotypes were selected based on the frequency in both the CERZOS germplasm and in nature (Carballo, Zappacosta, Selva, et al., 2021). Even though the number of 4x genotypes was higher than for other ploidy levels analyzed, their greater representation increased statistical power and avoided the detection of genotype‐specific genes related to other traits, such as reproductive mode. Overall, our data indicate that polyploidization does not dramatically affect the transcription of leaf protein‐encoding genes in *E. curvula* tetraploid genotypes, despite its clear effect on the epigenetic status (Carballo et al., 2024; Ochogavía et al., 2009). In contrast to similar transcriptomic studies conducted in *Medicago sativa* under normal and stress conditions (Santoro et al., 2025; Santoro et al., 2025), we found only a small number of genes with the same expression levels in tetraploids as in their diploid counterparts. Stochastic differences in the number of DEGs were observed among apomictic 4x genotypes, with Tanganyka USDA showing the greatest number of DEGs. On the contrary, a high proportion of DEGs was recorded at increasing ploidy levels. We assume that genotype‐dependent responses could exist within the 4x genotypes, whereas the higher number of DEG detected at increasing ploidy levels likely reflects a combined effect of increased genome copy number, the genetic background found at higher ploidies, and the number of genotypes used. Notably, the ratio between up‐ and down‐regulated genes was shifted at the highest ploidy level (8x) compared to the 2x counterpart. This pattern is consistent with previous observations in *Arabidopsis*, where increasing ploidy alters the balance of transcriptional regulation through dosage compensation mechanisms that buffer the effects of additional gene copies (Song et al., 2020). We also identified a core set of 443 Ploidy_vs_2x DEGs consistently expressed in a polyploidy‐sensitive manner. It is worth noting that only 35,440 transcripts out of the initial 54,589 (35,440/54,589 = 0.649; 65%) were retained, thus indicating that many genes are not being expressed in leaf tissue. We propose an interpretation of the transcriptomic patterns found at each ploidy level in Table 1. While a low number of DEGs was observed at the 4x level, an increasing number emerged at higher ploidy levels. These results may suggest a stochastic difference among 4x genotypes, whereas a combination of genomic buffering and genetic background variation likely reflects the high number of genes at increasing ploidy.

**TABLE 1:** Interpretation of different transcriptional responses in *E. curvula* at different ploidy levels.

Group	Transcriptional trends of unique DEGs			Interpretation	Biological meaning
	Total number	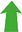	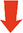		
4x_vs_2x	42	23	19	Genetic background variations among 4x genotypes, which in turn reduce the number of genes with the same expression patterns	Only a few genes involved with secondary metabolism and RNA/protein homeostasis were retrieved.
6x_vs_2x	1161	759	402	Transcriptomic reprogramming at increasing ploidy levels due to both genome buffering and genetic background	Putative genes related to stress responses, chromatin remodeling, hormonal regulation, ubiquitin‐proteasome system, growth and development were upregulated. Forage‐related genes associated with reduced forage quality and digestibility were found to be up‐regulated.
7x_vs_2x	1197	702	495		
8x_vs_2x	1518	755	763	Partial dosage compensation was observed at the highest ploidy level	
Ploidy_vs_2x	433	334	96	Core set of genes exhibiting the same pattern of expression levels in polyploidy genotypes compared to the 2x counterpart	

Abbreviation: DEGs, differentially expressed genes.

To quantify the proportion of gene expression variance that is attributed to ploidy and genotype, we fitted a linear mixed model using the variancePartition (Hoffman & Schadt, 2016). The results indicate that, although genotype contributes significantly to expression variability, ploidy:genotype interaction contributes significantly to expression variability, none of the factors alone analyzed here show a high percentage of the variance explained. This is expected in large and diverse datasets, such as those with different genomic backgrounds, ploidy levels, and reproductive modes. Analysis of GO terms revealed qualitatively and quantitatively distinct responses across ploidy levels, underscoring the presence of categories associated with distinct cellular compartments, molecular functions, and stress‐related pathways. Further analysis of the transcriptome using pathway enrichment revealed pathways uniquely modulated at each ploidy level. Interestingly, “metabolic pathways” and “flavonoid biosynthesis” pathways were found to be in common at higher ploidy levels (6x, 7x, and 8x) versus 2x. The most notable finding was the discovery of “Mismatch repair,” “Homologous recombination,” and “DNA replication” pathways, which were exclusively identified at the highest 8x ploidy level. These results suggest that polyploidization may activate conserved DNA repair and replication mechanisms in response to increased genomic instability. Similarly, Yin et al. (2021) reported a significant enrichment of these metabolic pathways in synthetic polyploid *Brassica napus* lines. MapMan analysis further revealed ploidy‐dependent reprogramming of flavonoid and terpenoid biosynthesis, photosynthetic processes, carbohydrate and lipid metabolism, hormone signaling, oxidative stress response, chromatin remodeling, RNA biosynthesis, and protein homeostasis. The analysis of the functional core set of 443 Ploidy_vs_2x genes consistently sensitive to ploidy level revealed the upregulation of genes related to ubiquitination pathway, hormone regulation, cell wall remodeling, and terpenoid biosynthesis. In particular, the upregulation of *GALT29A* is expected to influence cell‐wall architecture and organization by modifying arabinogalactan‐proteins (*AGPs*) (Dilokpimol et al., 2014), which, in turn, can interact with both pectin and hemicellulose to form cross‐linked networks. Moreover, the upregulation of four genes (*SUMO*, *FBL*, *DSK2*, and *DAR*) associated with the ubiquitin‐proteasome system clearly indicates how polyploid genotypes activate ubiquitin‐responsive genes to enhance tolerance to environmental stress conditions (Lyzenga & Stone, 2012; F. Xu & Xue, 2019). These findings may suggest a trade‐off whereby polyploids may enhance cell wall integrity and environmental resilience at the expense of forage digestibility.

We further identified relatively large ploidy‐specific differences in the patterns of TF families at higher ploidy levels. Among these, the most represented TF genes belong to the *FAR1* family, known to be involved in plant growth and development (Ma & Li, 2018). Many TF genes directly involved in chromatin remodeling and epigenetic regulation were differentially expressed at higher ploidy levels. Consistently, Carballo et al. (2024) reported that polyploid *E. curvula* genotypes displayed more differentially methylated genes than their diploid counterpart, highlighting the impact on epigenetic regulation under ploidy changes. Therefore, increasing ploidy levels may preserve genome integrity by reinforcing 5‐methylcytosine‐mediated silencing of transposable elements (Deniz et al., 2019; Zhang et al., 2018). Mining of hormone‐related TF genes, we found the upregulation of two *ERF* genes and one *B3 domain‐containing protein* *REM8* gene at the 6x level, while auxin‐ and cytokinin‐related genes were activated at the 8x level. However, we observed the upregulation of *ARR3*, known to act as a negative regulator of cytokinin signaling (To et al., 2004). Such equilibrium between synthesis and degradation may contribute to buffering cytokinin responses, ensuring that hormones accumulate to reach a *plateau* level before activating localized stress‐related processes (Roeder et al., 2022). In addition, a positive regulation of gibberellin biosynthesis was shared across higher ploidy levels, supporting the fundamental role of hormonal regulation in enhancing stress tolerance in polyploids (Van Hieu, 2019). Ubiquitin‐related TF genes were specifically identified at both 7x and 8x levels, confirming the role of proteasomal degradation of target proteins in polyploid plants (Martelotto et al., 2005). Consistent with this finding, Selva et al. (2020) reported differential expression of ubiquitination‐related genes associated with the transition from apomictic to sexual reproductive modes in *E. curvula* genotypes under water‐stress conditions. Interestingly, at the 8x level, we detected the upregulation of TF genes *PDF2*, *KNAT1*, *KNAT6*, and *YABBY1*, which are implicated in protoderm formation, embryo patterning, meristem maintenance, and organ development (Belles‐Boix et al., 2006; Ogawa et al., 2015; Truernit & Haseloff, 2008; Zhang et al., 2020). These findings further support the role of polyploidization in generating cellular‐level novelty, likely reflecting a dosage‐dependent response to increased ploidy (Doyle & Coate, 2019).

Characterization of co‐expression network analysis revealed modules that were correlated in a ploidy‐dependent fashion. Searching for those modules consistently correlated at higher ploidy levels, we observed a presumably increased biomass recalcitrance and reduced forage digestibility, mainly due to the upregulation of putative lignin‐biosynthetic genes (*HST*, *CAD7*, and *BGLU46*) together with the upregulation of the *WAT1*‐related protein (*WAT1*). Notably, Carballo, Zappacosta, Marconi et al. (2021) showed that three *WAT1* genes were found to be methylated at the 6 mA context in *E. curvula* apomictic genotypes. Moreover, the upregulation of wall‐remodeling hydrolases (*XTH32*, *BGAL14*, *BGLU10*, and *BGLU46*) together with decreased expression of pectin backbone lyase (*Rhamnogalacturonate lyase* *F21M12.28*) and a debranching enzyme (*alpha‐L‐arabinofuranosidase 2*) indicated a lowered enzymatic accessibility. Our data align with recent studies reporting that the downregulation of shikimate hydroxycinnamoyl transferase improved forage digestibility in alfalfa plants (Shadle et al., 2007). Recently, it has been reported that heightened lignification contributes to biotic and abiotic stress tolerance in grasses (Peracchi et al., 2024). Consistent with our findings, several stress‐responsive genes were specifically upregulated at higher ploidy levels, especially those involved in biotic and abiotic stress response. These results may corroborate a trade‐off in favor of stress tolerance over forage digestibility, further supporting the pivotal role of polyploidy in conferring abiotic and biotic stress tolerance (Tossi et al., 2022). Epigenetic‐responsive genes associated with DNA methylation (*NRPD1*, *FDM1*, and *MBD13*), histone modifications (*EZA1* and *AL2*), and epitranscriptomic regulation (*ECT3*) were also retrieved, indicating that a remodulation of the chromatin state occurred at higher ploidy levels in *E. curvula* (Carballo et al., 2024; Ochogavía et al., 2009).

Integrated network analysis was conducted to predict novel switch genes associated with polyploidy within those modules consistently correlated with ploidy‐sensitive expression patterns. As previously reported in other studies (Paci et al., 2017; Palumbo et al., 2014), switch genes have been proposed as key master regulators characterized by a marked negative correlation with the expression profiles of neighboring genes in the network. By exploring these master regulators involved in the transition from diploid to polyploid, we found that a predicted member of the *GH9B* subfamily of endo‐β‐1,4‐glucanases, a group of enzymes implicated in improving saccharification efficiency through cellulose microfibril modification (Buchanan et al., 2012; Huang et al., 2019), was downregulated. This finding is consistent with previous studies showing a positive correlation between endoglucanase activity and improved NDF degradability of alfalfa hay and corn silage (Eun & Beauchemin, 2007; Eun et al., 2007). Functional studies will be conducted to validate the role of these genes associated with important agronomical traits.

## 5 CONCLUSIONS

This study delves into the transition from diploid to polyploid state in *E. curvula*. Differential expression analyses revealed that the number of DEGs increased with ploidy level. Ploidy‐dependent transcriptional responses were observed. Epigenetic‐responsive genes associated with DNA methylation and epigenomic changes were also identified, suggesting that chromatin remodeling occurred at higher ploidy levels. These results suggest that *E. curvula*, in response to increased ploidy, brings about new nucleotypic effects in favor of stress tolerance and, presumably, decreases forage digestibility. Ploidy‐sensitive genes indicated an upregulation of genes encoding for ubiquitination pathway, hormone regulation, cell wall remodeling, terpenoid biosynthesis, and chromatin remodeling. Integrating network analysis revealed candidate target genes negatively associated with saccharification efficiency, paving the way for future genome editing experiments. Finally, different physiological traits such as FDA, FDN lignin, and protein will be collected from the same genotypes to accurately associate the gene expression to forage quality. Overall, our results provide valuable insights into the molecular mechanisms underlying polyploidy in *E. curvula*, with implications for breeding strategies aimed at balancing stress tolerance and improving biomass digestibility.

## AUTHOR CONTRIBUTIONS

**D. F. Santoro**: Data curation; formal analysis; methodology; software; validation; visualization; writing—original draft; writing—review and editing. **J. Carballo**: Data curation; formal analysis; methodology; software; validation; visualization; writing—review and editing. **M. C. Pasten**: Methodology; validation; writing—review and editing. **C. A. Gallo**: Data curation; formal analysis; methodology; software; validation; visualization; writing—review and editing. **E. Albertini**: Conceptualization; funding acquisition; investigation; methodology; project administration; supervision; writing—original draft; writing—review and editing. **V. Echenique**: Conceptualization; funding acquisition; investigation; methodology; project administration; resources; supervision; writing—original draft; writing—review and editing.

## CONFLICT OF INTEREST STATEMENT

The authors declare no conflicts of interest.

## Supporting information




**Supplemental Figure S1**: Hierarchical clustering map for normalized counts obtained using DESeq2, including principal component analysis (PCA) and phylogenetic relationships among *E. curvula* genotypes at different ploidy levels.

**Supplemental Figure S2**: Variance partitioning attribute to genotype within ploidy, ploidy and genotype through a linear mixed model.

**Supplemental Figure S3**: WGCNA modules identified by the k‐means clustering analysis applied to ploidy‐responsive genes.

**Supplemental Table S1**: *E. curvula’*s genotypes with different ploidy levels and reproductive modes used in this study.

**Supplemental Table S2**: Summary statistics of the RNA sequencing.

**Supplemental Table S3**: List of differentially expressed genes (DEGs) identified in all comparisons, including the associated log_2_FoldChange, adjusted *p*‐value, and the predicted *Arabidopsis* orthologs.

**Supplemental Table S4**: List of GO terms enriched within each group.

**Supplemental Table S5**: List of genes identified by Mapman software at each ploidy level.

**Supplemental Table S6**: Ploidy‐responsive transcription factor families.

**Supplemental Table S7**: List of module eigengenes identified at each ploidy level and putative hub genes identified by the SWIM tool.

## Data Availability

The datasets presented in this study can be found in online repositories: https://www.ncbi.nlm.nih.gov/, BioProject: PRJNA1338197.
