# Supplementary material for: Traversing the effects of ploidy changes in different Eragrostis curvula genotypes through high‐throughput RNA sequencing
Source: Plant Genome. 2026 Mar 28;19(2):e70227. doi: 10.1002/tpg2.70227 (PMC13032165; doi:10.1002/tpg2.70227)
Supplement: Supplementary file 1 [file TPG2-19-e70227-s002.pdf]

Authors: Danilo Fabrizio Santoro, José Carballo, Maria Cielo Pasten, Cristian Andres Gallo, Emidio Albertini and Viviana Echenique.

Manuscript title: Traversing the effects of ploidy changes in different *Eragrostis curvula* genotypes through high-throughput RNA sequencing.

Number of pages: 44, number of figures: 5, number of tables: 1

Table S2. Summary statistics of the RNA sequencing. The average RIN was 7.3

Sample	Total reads	Mapped reads	Unmapped reads	% of mapped reads
Bahiense 1	66801062	65281222	1519840	97.72
Bahiense 2	953103057	935725319	17377738	98.18
Bahiense 3	71023335	69357024	1666311	97.65
Don Eduardo 1	50582319	48577507	2004812	96.04
Don Eduardo 2	76913844	72834186	4079658	94.70
Don Eduardo 3	107804647	101958834	5845813	94.58
Don Juan 1	92891637	87643816	5247821	94.35
Don Juan 2	158509293	152621372	5887921	96.29
Don Juan 3	123418437	117427422	5991015	95.15
Don Pablo 1	60964282	57344956	3619326	94.06
Don Pablo 2	72792330	66433639	6358691	91.26
Don Pablo 3	73616706	69736373	3880333	94.73
Ermelo 1	113010844	109782157	3228687	97.14
Ermelo 2	102572450	99937483	2634967	97.43
Ermelo 3	105234481	102914882	2319599	97.80
Morpa 1	122723697	119175821	3547876	97.11
Morpa 2	290490713	284911234	5579479	98.08
Morpa 3	127990202	124401337	3588865	97.20
OTA-S 1	136221331	133538413	2682918	98.03
OTA-S 2	145647026	141703269	3943757	97.29
OTA-S 3	108310553	105471083	2839470	97.38
Tanganyika INTA 1	87133704	84892698	2241006	97.43
Tanganyika INTA 2	109168768	106838734	2330034	97.87
Tanganyika INTA 3	139487877	136557201	2930676	97.90
Tanganyika USDA 1	467733283	452282542	15450741	96.70
Tanganyika USDA 2	143682047	139321015	4361032	96.96
Tanganyika USDA 3	136563481	132229890	4333591	96.83
Victoria 1	149381421	146870901	2510520	98.32
Victoria 2	95996647	94817023	1179624	98.77
Victoria 3	132184200	130184132	2000068	98.49
