# Supplementary material for: Traversing the effects of ploidy changes in different Eragrostis curvula genotypes through high‐throughput RNA sequencing
Source: Plant Genome. 2026 Mar 28;19(2):e70227. doi: 10.1002/tpg2.70227 (PMC13032165; doi:10.1002/tpg2.70227)
Supplement: Supplementary file 2 [file TPG2-19-e70227-s007.pdf]

Authors: Danilo Fabrizio Santoro, José Carballo, Maria Cielo Pasten, Cristian Andres Gallo, Emidio Albertini and Viviana Echenique.

Manuscript title: Traversing the effects of ploidy changes in different *Eragrostis curvula* genotypes through high-throughput RNA sequencing.

Number of pages: 44, number of figures: 5, number of tables: 1

Table S1. *E. curvula*'s genotypes with different ploidy levels and reproductive modes used in this study.

Genotype	Ploidy	Reproductive mode
Victoria	Diploid 2n=2x=20	Sexual
OTA-S	Tetraploid 2n=4x=40	Sexual
Tanganyika USDA	Tetraploid 2n=4x=40	Full apomict
Tanganyika INTA	Tetraploid 2n=4x=40	Facultative apomict
Ermelo	Tetraploid 2n=4x=40	Facultative apomict
Morpa	Tetraploid 2n=4x=40	Facultative apomict
Bahiense	Tetraploid 2n=4x=40	Facultative apomict
Don Eduardo	Hexaploid 2n=6x=60	Facultative apomict
Don Pablo	Heptaploid 2n=7x=70	Facultative apomict
Don Juan	Octoploid 2n=8x=80	Facultative apomict
