# Supplementary material for: Traversing the effects of ploidy changes in different Eragrostis curvula genotypes through high‐throughput RNA sequencing
Source: Plant Genome. 2026 Mar 28;19(2):e70227. doi: 10.1002/tpg2.70227 (PMC13032165; doi:10.1002/tpg2.70227)
Supplement: Supplementary file 2 — Supplemental Figure S2: Variance partitioning attribute to genotype within ploidy, ploidy and genotype through a linear mixed model. [file TPG2-19-e70227-s010.pdf]

Authors: Danilo Fabrizio Santoro, José Carballo, Maria Cielo Pasten, Cristian Andres Gallo, Emidio Albertini and Viviana Echenique.

Manuscript title: Traversing the effects of ploidy changes in different *Eragrostis curvula* genotypes through high-throughput RNA sequencing.

Number of pages: 44, number of figures: 5, number of tables: 1

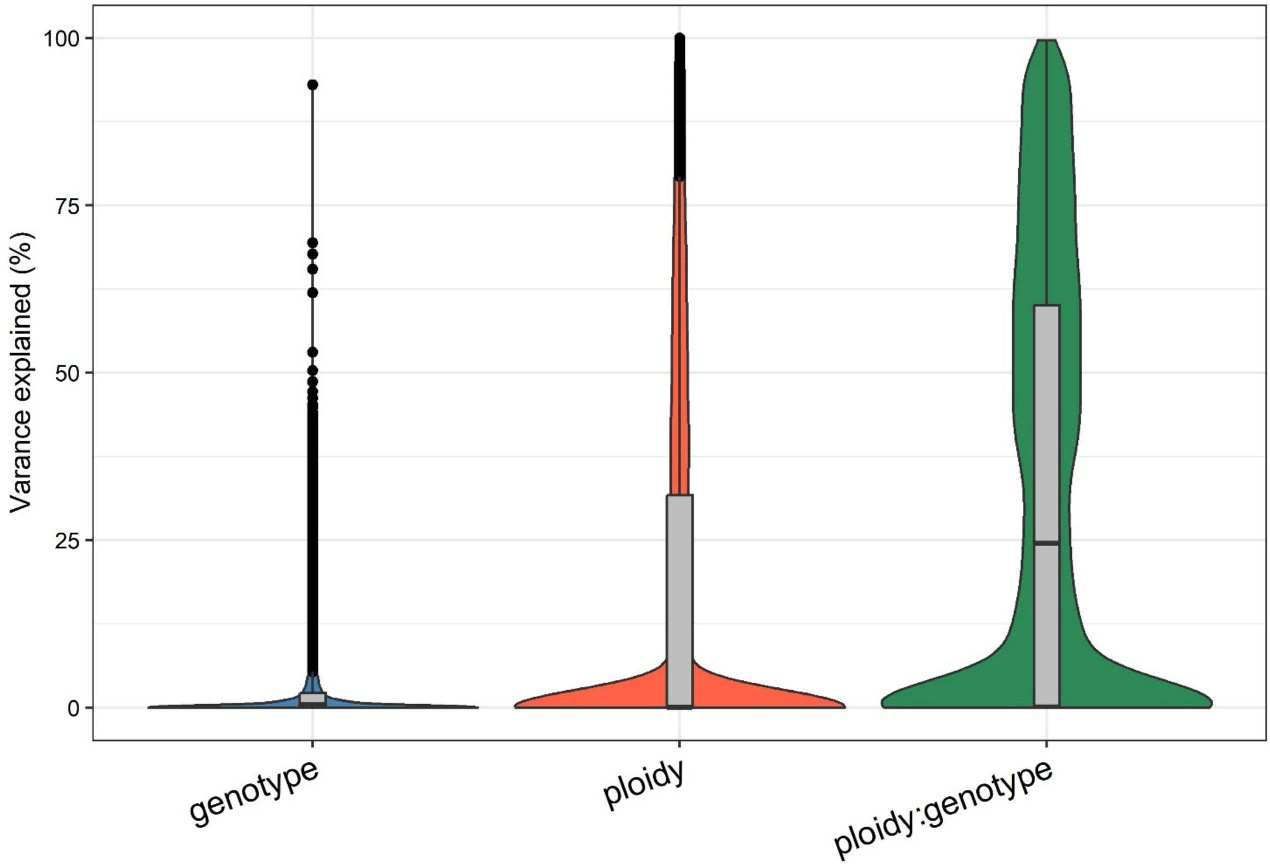

Figure 2. Variance partitioning of gene expression levels using a linear mixed model with random effects for ploidy, genotype, and ploidy:genotype. The violin/boxplot displays the distribution of variance fractions across expressed genes.
