# Supplementary material for: Traversing the effects of ploidy changes in different Eragrostis curvula genotypes through high‐throughput RNA sequencing
Source: Plant Genome. 2026 Mar 28;19(2):e70227. doi: 10.1002/tpg2.70227 (PMC13032165; doi:10.1002/tpg2.70227)
Supplement: Supplementary file 3 — Supplemental Figure S3: WGCNA modules identified by the k‐means clustering analysis applied to ploidy‐responsive genes. [file TPG2-19-e70227-s008.pdf]

Authors: Danilo Fabrizio Santoro, José Carballo, Maria Cielo Pasten, Cristian Andres Gallo, Emidio Albertini and Viviana Echenique.

Manuscript title: Traversing the effects of ploidy changes in different *Eragrostis curvula* genotypes through high-throughput RNA sequencing.

Number of pages: 44, number of figures: 5, number of tables: 1

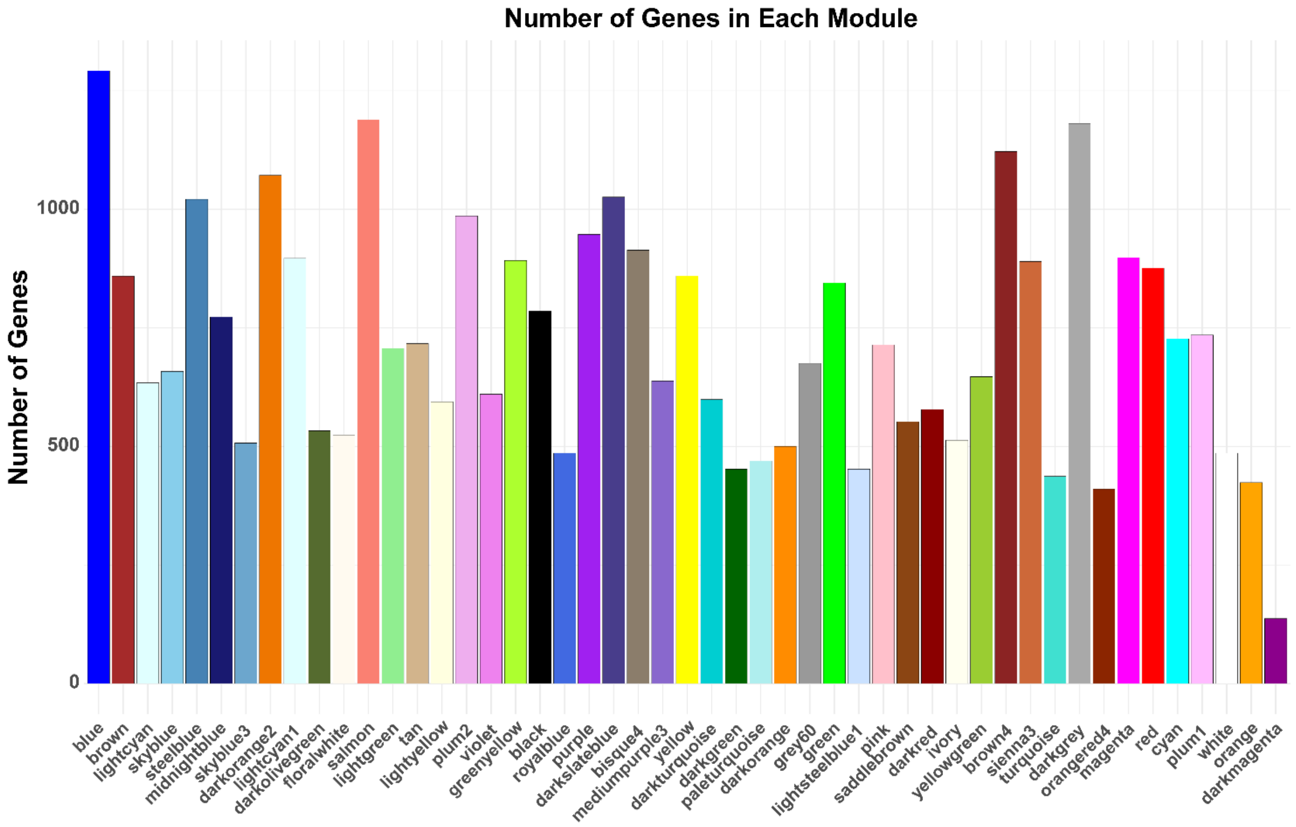

Figure S32. WGCNA modules identified by the k-means clustering analysis applied to ploidy-responsive genes.
